# Raman Spectroscopy: A Tool for Molecular Fingerprinting of Brain Cancer

**DOI:** 10.1021/acsomega.3c01848

**Published:** 2023-07-27

**Authors:** Sivasubramanian Murugappan, Syed A. M. Tofail, Nanasaheb D. Thorat

**Affiliations:** Department of Physics, Bernal Institute and Limerick Digital Cancer Research Centre (LDCRC) University of Limerick, Castletroy, Limerick V94T9PX, Ireland

## Abstract

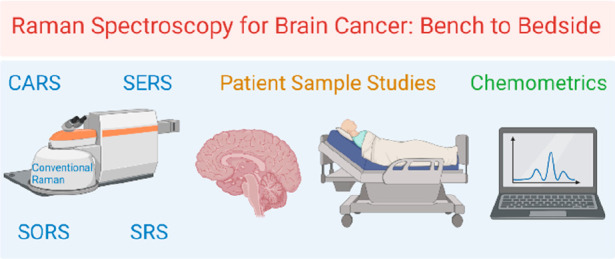

Brain cancer is one of those few cancers with very high mortality and low five-year survival rate. First and foremost reason for the woes is the difficulty in diagnosing and monitoring the progression of brain tumors both benign and malignant, noninvasively and in real time. This raises a need in this hour for a tool to diagnose the tumors in the earliest possible time frame. On the other hand, Raman spectroscopy which is well-known for its ability to precisely represent the molecular markers available in any sample given, including biological ones, with great sensitivity and specificity. This has led to a number of studies where Raman spectroscopy has been used in brain tumors in various ways. This review article highlights the fundamentals of Raman spectroscopy and its types including conventional Raman, SERS, SORS, SRS, CARS, etc. are used in brain tumors for diagnostics, monitoring, and even theragnostics, collating all the major works in the area. Also, the review explores how Raman spectroscopy can be even more effectively used in theragnostics and the clinical level which would make them a one-stop solution for all brain cancer needs in the future.

## Introduction

Raman Spectroscopy is one of those very few techniques which is label-free (other major ones being NMR, XPS, UV–vis, FTIR, mass spectroscopy), nondestructive, and cost-effective among those that can give a molecular profile of the samples. Numerous research groups are working on improving the conventionally used Raman instruments, integrating them with other instruments to utilize as a multimodal tool, and changing/enhancing parameters to increase performance efficiency, portability, affordability, etc. This has led to various Raman spectroscopy types namely resonance Raman, surface-enhanced Raman (SERS), spatially offset Raman (SORS), confocal Raman microspectroscopy, etc. They have been used in various industries including forensics, pharma, food, etc., especially in quality control. Raman being nondestructive is also very well suited for biological sample analysis too. This potentiates its capability to act as a disease diagnostics and monitoring instrument. Lately, they have been modified and integrated to be used in treatment too.

Raman Spectroscopy works based on the Raman effect where the inelastic scattering of photons results in their movement from ground vibrational energy states to virtual energy states and back. But if they stop at a higher or lower vibrational energy state than where they started, it is called Stokes and anti-Stokes Raman scattering, respectively.^1^ This when recorded for different samples varies according to their composition which is digitally represented in the form of peaks based on the intensity and frequency variations observed, helping in the identification of the sample composition. This helps Raman spectroscopy recognize and distinguish cell lines and tissue samples both in vivo and ex vivo. Moreover, within these groups too, they can classify based on the biochemical composition be it normal or tumorous, between different grades of cancer, etc.

Brain tumor is caused by the abnormal growth of cells in the brain region. Still, why a person gets a brain tumor is not well established but a few reasons for getting one include rare genetic conditions like von Hippel-Lindau disease, tuberous sclerosis, etc. Brain tumor usually occurs in the meninges or glial cells. In most cases, a meningioma tumor in the meninges is benign, while glial cells called glioma can be malignant. The tumors are classified into 4 grades with malignancy increasing with each grade. Normally, the grade 1 tumors are benign, in gliomas too. Grade 2 can/cannot be benign, whereas grades 3 and 4 are malignant. They are also categorized into primary and secondary brain cancers based on the site of origin with cancers starting in the brain itself considered to be primary and the brain metastases from other cancers as secondary.^2^

**Figure 1:**
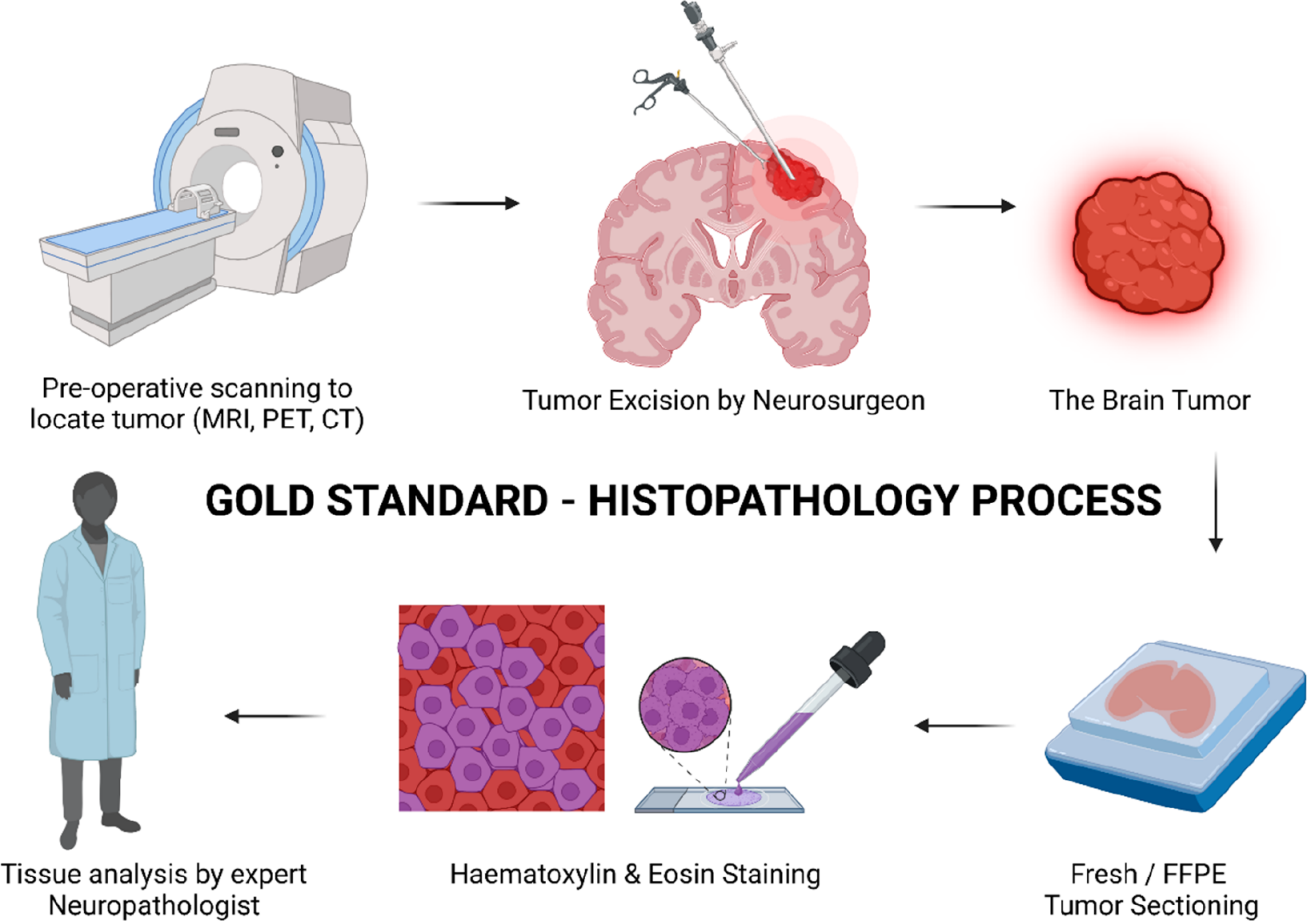
Schematic representation of the long and tedious histopathology process. The patients need to undergo initial scanning to identify the tumor. This is followed by a sample section which further undergoes multiple processing steps taking longer time and needs to be confirmed by a neuropathologist which increases the chances of error. An error or partial removal of tumor leads to invasive surgery, again affecting the health and quality of life of patient. (Created with Biorender.com.)

**Figure 2:**
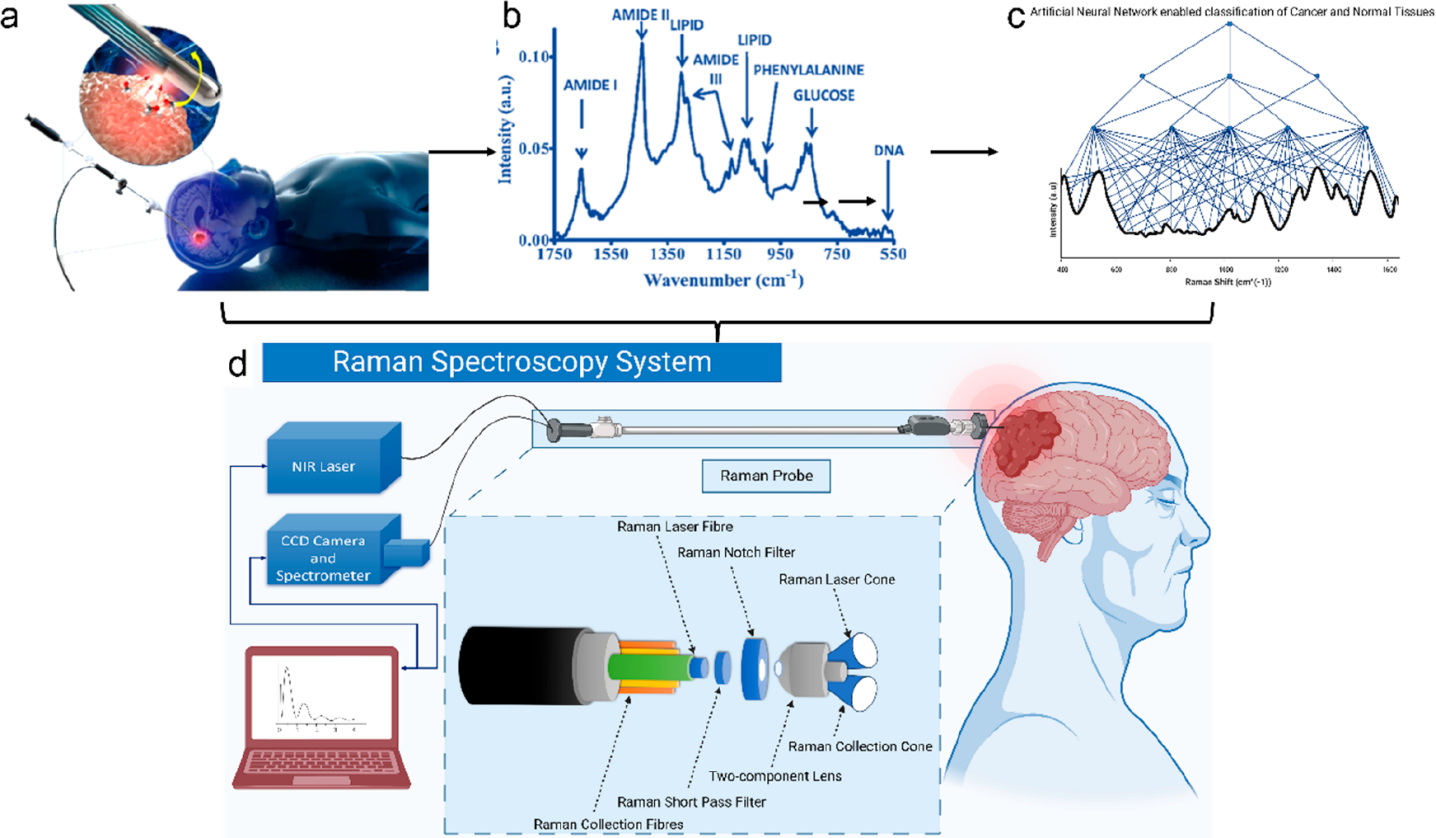
Schematic representation of the quick and reliable Raman spectroscopy process. Raman laser probe is used over the sample (a)^5^ which gives the Raman spectra (b).^6^ Reprinted with permission from ref (5). Copyright 2018 Nature. Reprinted from ref (6). Copyright 2011 American Chemical Society. This on processing using the computational model distinguishes between tumor and normal specimen (c). Overall system containing all these is the Raman spectroscopy setup (d). (Figure 2d was created with BioRender.com.)

There were 308,102 new cases and 251,329 fatalities due to brain and CNS cancers worldwide in 2020, according to the GLOBOCAN 2020 report.^3^ Also, brain cancer has one of the lowest 5% year survival rates among all other cancer types.^4^ These statistics and the complexity of brain tumors clearly signify the requirement for better diagnostics, monitoring, and treatment techniques. Here, Figure 1 shows the most followed histopathology process, while Figure 2 shows how Raman spectroscopy can, in comparison, be more helpful in all three scenarios discussed above. Thus, in this article, we will be reviewing the works which discuss the ability of Raman spectroscopy in the diagnosis, monitoring, and treatment (surgery) of brain tumors.

## 2 Molecular Fingerprinting of Brain Tumor

Riva et al. analyzed 3450 spectra from 63 glioma biopsy samples within 60 min of resection without any preprocessing. Analyzing of samples via Raman revealed 19 new shifts which have not been reported before representing calcification (975 cm^–1^), collagen (817 cm^–1^), heme content (743 cm^–1^), glycogen (941 cm^–1^), lipids (431, 776, 875, 968 cm^–1^), nucleic acids (498, 780, 825, 894 cm^–1^), and proteins (524, 933, 963, 1031, 1035, 1583, 1603 cm^–1^) (represented in Table 1).^7^ Kopec et al. suggested that the peaks obtained from aggressive brain tumors at wavenumbers 1004 cm^–1^ (proteins), 1156 and 1520 cm^–1^ (carotenoids), 1585 cm^–1^ (cytochrome), and 1444 and 1655 cm^–1^ (fatty acids) are potential biomarkers for oncological diagnosis.^8^ The major peaks related to low-grade glioma are proline/tyrosine (877, 852 cm^–1^) and choline/cholesterol (877 cm^–1^), while it was phenylalanine (1004, 1032 cm^–1^); tryptophan (1553, 1339 cm^–1^); amide III, collagen, and nucleic acid (1339 cm^–1^); and amide I, proteins, lipids, and nucleic acid (1659 cm^–1^) for high-grade glioma.^9^ The following peaks were the factors of differentiation between the two grades: C=O stretching (1858 cm^–1^), CH_2_ bending (1450 cm^–1^), amide III and CH_2_ deformation (1230–1360 cm^–1^), structural changes of phospholipid (1130 cm^–1^), and polysaccharides/amino acids (850 cm^–1^).^10^ The peak between 2889 and 2934 cm^–1^ (lipids and lipoproteins) had a lower intensity than the ones at 1667 cm^–1^ (collagen and amide) in cancer tissues and vice versa in normal tissues, which can be paved back to structural changes in tissue during the course of development into cancer where cell proliferation is high.^11^ It was also pinpointed by Iturrioz-Rodríguez et al. that the proliferation rate and mitochondrial content play a major role in cancer as the peaks of RNA/DNA and cytochrome C are increased in glioma cells.^12^

**Table 1:** Major Peaks of Brain Tissues in Raman Spectrum, with Their Tentative Assignments^7,15,16,12^

Raman shift (cm^–1^)	assignment
421	cholesterol
425	cholesterol
431	cholesterol/cholesterol ester
450	ring torsion of phenyl
457	proteins and cholesterol
474	glycogen and polysaccharides
478	polysaccharides
498	nucleic acids, characteristic for DNA
524	S–S disulfide stretching in proteins
540	n(S–S) stretching (amino acid cysteine)
544	phospholipids
545	cholesterol
547	cholesterol
596	phosphatidylinositol
597	melanin
607	phospholipids
612	cholesterol
620	C–C twist aromatic ring (Phe)
624	phenylalanine
640	C–S stretching of cystine
642	C–C twisting mode of tyrosine
667	C–S stretching of cystine, collagen
670	hemoglobin
683	nucleic acids, characteristic of DNA
699	phospholipids
700	C–O stretching
700	cholesterol and phospholipids
717	phospholipids, choline groups
719	C–N+ stretching of choline
727	nucleic acids, characteristic of DNA
750	oxygenated hemoglobin, cytochrome
757	protein (Trp), hemoglobin
780	O–P–O stretching of DNA, uracil-based ring breathing mode
782	DNA and/or RNA
817	stretching/collagen assignment
825	phosphodiester
826	Tyr, proline
826	O–P–O stretching of DNA and/or RNA
829	tyrosine
852	C–C stretching of tyrosine, collagen
853	tyrosine
857	protein (Tyr), collagen
875	phospholipids, choline groups
877	cholesterol
880	tryptophan, d(ring)
881	hydroxyproline and tryptophan (collagen); sterol ring stretch of cholesterol; asymmetric stretching of choline
883	(CH_2_) (protein assignment)
893–894	phosphodiesters (nucleic acids)
925	C–C bonds of the peptide backbone
926	C–C bond of the peptide backbone
928	amino acids proline and valine (protein band)
933	proline, hydroxyproline
934	C–C backbone (collagen assignment)
936	C–C stretching of Proline, valine, collagen
938	C–C stretching (Amide III) — protein
940	protein, collagen
941	glycogen
950	single bond stretching vibrations for the amino acids proline and valine and polysaccharides
958	stretching vibrations of PO_4_ in hydroxyapatite
961	cholesterol
963	protein assignments
968	lipids
976	melanin
977	tricalcium phosphate Ca_3_(PO_4_) calcification seen in schwannoma and necrosis
1002	phenylalanine, oxygenated hemoglobin
1003	γs(C–C) phenylalanine ring breathing mode
1031	phenylalanine
1035	collagen
1062	O–P–O stretch DNA and RNA, phospholipids, saturated fatty acids, cholesterol
1081	lipids
1086	brain phospholipids (C–C and C–O stretching)
1086	nucleic acid
1089	fatty acids phosphate backbone
1092	characteristic for DNA
1097	nucleic acid
1122	glycogen
1126	brain phospholipids
1127	cytochrome c
1128	C–C stretching vibrations, typical for proteins
1128	cholesterol and phospholipids
1129	fatty acids
1129	lipid γ(C–C)
1155	beta-carotene, C–C and C–N stretching of proteins
1159	carotenoids
1174	C–H deformation of proteins
1174	nucleic acid (cytosine, guanine)
1176	breathing mode of phenylalanine
1208	phenylalanine (protein)
1210	protein (Phe, Tyr)
1212	oxygenated hemoglobin
1225	hemoglobin
1247	collagen, protein (amide III)
1250	nucleic acid
1250	hemoglobin
1255	nucleic acids, characteristic of DNA
1255	amide III, typical for proteins
1266	major protein bands
1267	phospholipids, unsaturated fatty acids
1267	amide bands of protein backbones
1269	amide II and III
1269	brain phospholipids
1296	major protein bands
1296	cholesterol and phospholipids
1299	fatty acids
1313	lipids
1313	collagen
1333	proteins and nucleic acids
1337	aliphatic amino acids (C–H deformation), including tryptophan, nucleic acids; glycogen in necrosis
1340	tryptophan
1340	nucleic acids, characteristic of DNA
1342	aliphatic amino acids, including tryptophan, nucleic acids; glycogen
1370	nucleic acid
1376	nucleic acids, characteristic of DNA
1397	lipids
1397	CH_3_, CH_2_ deformation of collagen
1404	melanin
1420	nucleic acids, characteristic of DNA
1436	major protein bands
1439	proteins and lipids
1439	phospholipids, saturated fatty acids
1440	lipid δ(CH_2_)
1447	aliphatic amino acids
1450	protein (CH_2_/CH_3_)
1486	nucleic acids, characteristic for DNA
1521	phospholipids (sphingomyelin), carotenoids
1523	carotenoids
1546	oxygenated hemoglobin
1556	indole ring, tryptophan
1566	hemoglobin
1578	nucleic acid
1581	C–C stretch of protein, nucleic acids
1583	C–C stretch of protein, phenylalanine, nucleic acids
1585	hemoglobin
1586	cytochrome
1595	melanin
1603	cytosine, phenylalanine and tyrosine/oxygenated hemoglobinn
1605	oxygenated hemoglobin
1614	aromatic amino acids (protein); tyrosine and proline
1616	C–C stretching mode of tyrosine and tryptophan
1619	oxygenated hemoglobin
1623	hemoglobin
1657	lipids
1658	major protein bands
1660	protein and lipids
1661	amide II and III
1661	phospholipids, unsaturated fatty acids
1667	amide
1668	cholesterol
1735	cholesterol
1739	cholesterol ester
1225–1300	amide III
1580–1700	nucleic acid
1645–1675	amide

**Figure 3:**
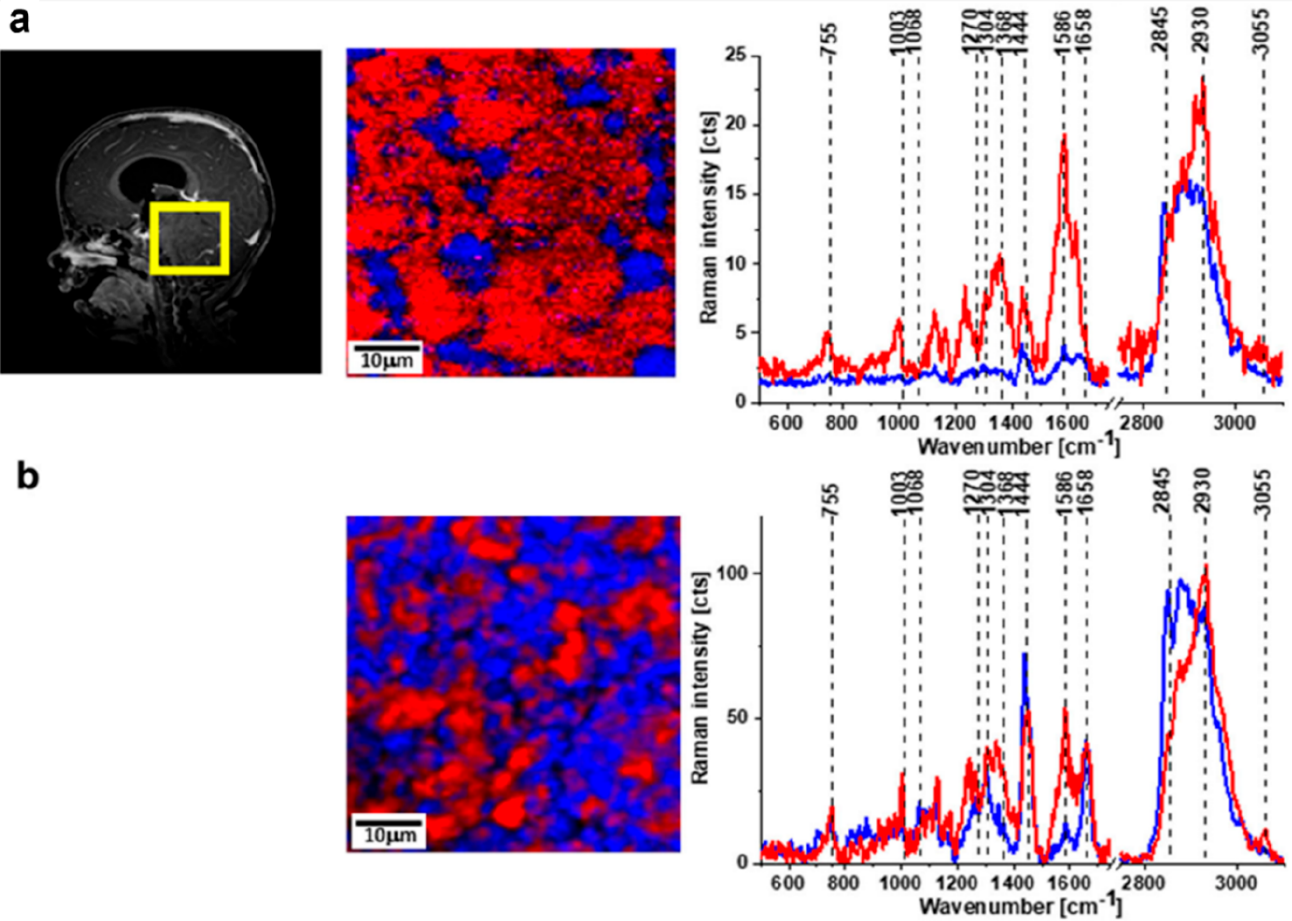
MRI, Raman image, and Raman spectra of normal brain tissues (a). Raman image and Raman spectra of medulloblastoma (b). Lipid (red) and proteins (blue). The decrease in the intensity of red color in Raman image (b) clearly demonstrates that the lipid levels decrease in tumor tissues which is affirmed by a high intensity blue peak (protein) and comparatively low intensity red peak (lipid).^13^ Reprinted with permission from ref (13). Copyright 2018 Springer.

The peak at 1586 cm^–1^, which represents C=C bending of phenylalanine and acts as a marker for malignancy, was also found to correspond to tyrosine phosphorylation apart from the amide III band shift to 1228 cm^–1^ from 1270 cm^–1^. The ratios of *A*_proteins/lipids_, *I*_1586/1444_, and *I*_2930/2845_, clearly higher in medulloblastoma tissue than that in normal ones, are clearly seen in Figure 3 establishing lower lipid content in cancer. The average area containing lipid content in tumorous tissue was 24%, while it was 58% in normal tissues.^13^ An increase in peak levels was significant in protein bands (2930 cm^–1^) of dense cancer cells. Also, the protein/lipid ratio saw a spike in dense cancer cells compared to normal but was not the same between infiltered and normal cells.^5^ Another study from the same group supports the previous literature showing enhanced conformational changes from α-helix to β-sheets on tumor progression and an increase in the lipid-to-protein ratio from 1.46 ± 0.02 in medulloblastoma to 1.99 ± 0.03 in normal brain tissues.^14^

A nonexhaustive yet comprehensive list representing the Raman peaks involved affecting the brain with their most evident biochemical groups obtained from different sources are given below in Table 1.

## 3 Raman Spectroscopy in Diagnosis

### 3.1 Solid Tissue Sample

Raman spectroscopy due to its ability to identify the biochemical changes precisely can be an excellent tool to diagnose cancer and all its subtypes with high accuracy. Iturrioz-Rodríguez and group collected samples from four male glioma patients and tried to use Raman spectroscopy for differentiating the healthy astrocytes from the cancerous region, especially determining the tumor margin properly. The outcome concludes that the band ranging between 1000 and 1300 cm^–1^ is sufficient to predict cancer cells with an accuracy of 92.5%.^12^ In another study, a proof of concept was developed by Ralbovsky and colleagues for deep-ultraviolet Raman spectroscopy. Deep-UV could be a potential option against conventional Raman spectroscopy involving visible or IR spectra because of UV radiation’s ability to absorb and excite at the same wavelengths, resulting in the enhanced resonance of Raman signals. In this work, they used a UV wavelength of 198 nm (deep-UV) against NOD-SCID mice having brain cancer inflicted with breast cancer cells. The UV exposure was fixed at 120 J/cm^2^ following the guidelines of the International Commission on Non-Ionizing Radiation Protection (ICNIRP).^17^

In a similar study, Raman spectroscopy was used to discriminate the glioblastoma, metastasis, and normal groups. The group used the ratio between the peaks 721 cm^–1^, the symmetric C–N stretch of choline, and 782 cm^–1^, the uracil/cytosine ring breathing of nucleotides, to arrive at the sensitivity and specificity levels. It was inferred that the sensitivity and specificity levels remained above 85% in all three cases.^18^

The study by Zhou et al. revealed that cancer tissues showed a reduction in the intensity of the fatty acid-rich resonance Raman (RR) peak at 2885 cm^–1^ and the taller peak of the protein band at 2931 cm^–1^ compared to 2885 cm^–1^ unlike in healthy normal tissues. Also, the peak shift from 1358 cm^–1^ in low-grade glioma to 1378 cm^–1^ in high-grade glioma discloses the domination of deoxy-hemoglobin in low and oxy-hemoglobin in high-grade glioma, thereby helping in hypoxia and necrosis monitoring. Also, the shift in peaks of amide I and amide III in normal and grade IV glioma tissue suggests a structural conformation change from α-helix to β sheets. The predicted reason for the change is the mutation of tryptophan W104. The team used a confocal micro-Raman spectrometer with an excitation wavelength of 532 nm, taking 510 RR spectra from over 121 subjects, and the results were also compared with the results of the histopathological examination which is the WHO gold standard.^19^

Till now, the predominant way of interpreting Raman signals between normal and cancer tissues is by their peak intensity and shift in wavenumbers. In this study, Kaushik et al. studied the full width at half-maximum (fwhm) values to diagnose cancer based on the analogy in semiconductors that the most sensitive parameter of Raman spectra is the subtle variation of width confirmed by the effect of acceptor–donor interactions and quantum size. The hypothesis was established by the fact that the team found a decrease in fwhm from 8 to 5 cm^–1^ (37%) that was seen in brain cancer samples against the normal ones at 1001 cm^–1^ (protein). Similar results were also obtained for 1349 cm^–1^ (nucleic acid) and 1379 cm ^–1^ (lipids). They showed a 13.8% increase and a 2% decrease, respectively.^20^

Apart from chemometrics, there are a lot of parameters in terms of the Raman spectroscopy instrument which can be optimized to get better diagnosis.^21^ A study on one such parameter was conducted by Leblond and group. They varied spectra intensity qualitatively (visual) and quantitative quality factors classifying spectra into low and high. These low and high intensity spectra were applied on 44 brain cancer patients using a hand-held Raman probe. The results after chemometrics studies showed an increase of 20% sensitivity and 12% specificity in high spectral images to that of its counterpart.^22^

### 3.2 Liquid Sample

A biosensor was developed by Malsagova and team to check the prediction capacity of Raman for brain cancer from human plasma samples. The “silicon-on-insulator” (SOI) nanowires (NW) they created were surface-modified with an analog of miRNA-363 (a brain cancer marker). These were tested in a buffer solution to know the maximum efficiency of the developed system and to have an initial quantity to proceed with for the plasma samples. The system used for plasma samples was surface-modified with miRNA-363 itself. Overall, the sensor was sensitive, starting from a concentration level of 3.3 × 10^–17^ M.^23^

## 4 Raman Spectroscopy in Monitoring

This unique study used Raman spectroscopy to monitor responses of T-cells and monocytes in tumor-conditioned media of glioblastoma by introducing CD73 and EMT activator ZEB1, regulators of cancer cell immunogenicity. The results were analyzed using multivariate analysis tools, PCA for unsupervised and LDA and SVM for supervised analysis. The results obtained were matched with that of flow cytometry. Both results indicated similar changes in the immunomarkers. The major changes were seen in CD209, CD64, CD4, CD8, and CD11c. From the results, it can be interpreted that T-cells and monocytes are influenced and are differentiated into mixed populations of anti- and pro-tumorogenic macrophages and dendritic cells by ZEB1 and CD73, respectively.^24^

Surmacki studied the effects of γ radiation on Daoy cells (medulloblastoma) by both labeled (oxidative stress and metabolic activity) and label-free (Raman spectroscopy) methods. The work uses therapeutic doses of irradiation of 2 Gy and 10 Gy. The spectral analysis indicated the degradation of proteins and membranes after treatment with 2 Gy γ radiation. Further, data analysis shows an average sensitivity and specificity of 80.35% and 79.65%, respectively. Thus, this combination of Raman spectroscopy along with other histological techniques can be used as a monitoring tool for radiation-treated medulloblastoma patients in the future after deeper study on the same.^25^

Raman spectroscopy will be used more often in the case of monitoring than diagnosis or treatment. SORS with optimal offset values would be a great choice as they can capture the signals inside the skull from outside, thereby contributing to the patient’s good by maintaining noninvasiveness.

## 5 Raman Spectroscopy in Surgery

A Raman imaging study carried out over a period of 1.5 years in 209 patients with different types of brain cancer was able to identify recurring glioblastoma with 100%, primary with 94% and glioma metastases with 90% accuracy. Similarly, for oligodendroglioma, astrocytes, and their IDH1 mutant versions, the accuracies stood at 90%, 86%, and 81%, respectively.^26^ Another work by Livermore and group studies the ability of Raman to classify brain cancer based on their genetic subtypes. This experiment was from fresh samples collected from 62 patients having IDH wildtype and IDH mutated astrocytoma and oligodendroglioma. Apart from these, the group also has cryosectioned, FFPE, and LN-18 (IDH-wildtype and mutated parental) glioma cell lines. The results of all these samples were further authenticated by genetic sequencing and immunohistochemistry. Quantitatively, the sensitivity and specificity for predicting among the 3 tissue types were 79–94% and 90–100%, respectively. The overall time for all types of classification and prediction was within 15 min per sample.^27^ Uckermann and team explored the feasibility of using Raman Spectroscopy for identifying IDH1 mutation glioma tissue samples. Overall, there was a surge in DNA-related spectral bands while that of lipids decreased. Also, variations in spectral bands corresponding to protein were seen between IDH1 wild type and IDH1 mutant gliomas. The classification was finally done only based on 5 bands (498, 826, 1003, 1174, and 1337 cm^–1^) and the accuracy was 89%.^15^

Auner et al. analyzed 64 samples either fresh or frozen from 28 pediatric patients to differentiate between tumors and their grades. The tissues had accuracy levels above 90%, while their differential sensitivity and specificities for low and high-grade ependymomas were 100% and 96% and between normal and low-grade glioma it was 91.5% and 97.8%, respectively.^28^ The same team published the results of a comprehensive 6-year study on pediatric patients with solid tumors. The work used Raman to detect brain tumors among other types. A training set based on PCA-DFA gave 95.1% accuracy, while the testing group gave 88.9%. The team also tested the algorithm in a generalized database and found 85.5% accuracy in spotting brain cancer. The histopathology verifications were performed by pediatric pathology specialists.^29^ One more team also worked on pediatric patients. In their work, ex vivo brain tissues of 29 pediatric patients were imaged using Raman. The images were trained via machine learning algorithms to discriminate between normal and tumorous brain tissues and between normal and low-grade glioma (LGG). The accuracy was achieved at around 85% in both cases.^30^

**Figure 4:**
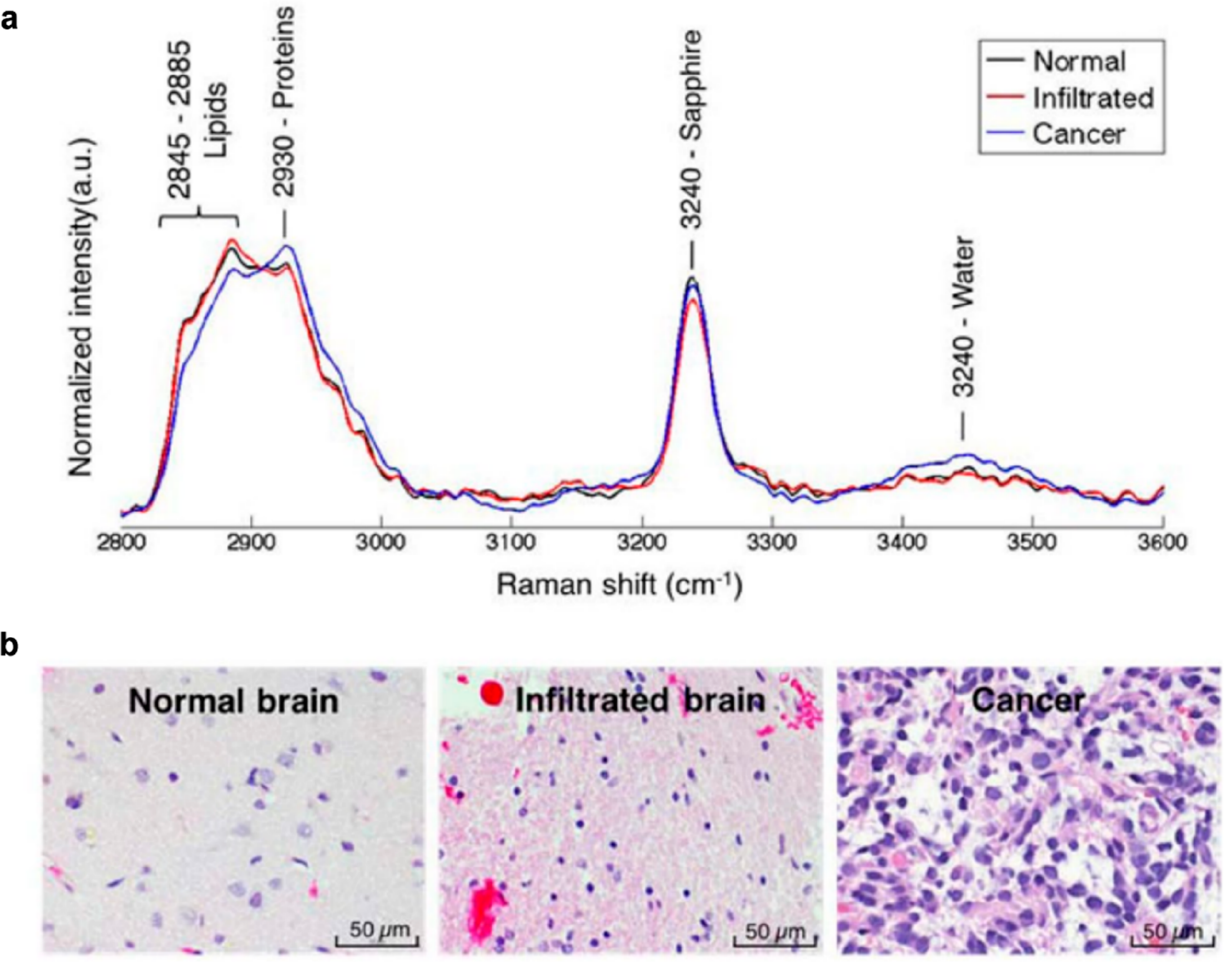
Raman spectra of normal, infiltrated, and cancer tissues (a). H&E staining of normal, infiltered, and cancer tissues (histopathology) (b). Cancer spectra (blue) in panel (a) distinctly shows a peak between 2845 and 2885 cm^–1^, representing its low lipid content in line with the literature studies which impart that a high protein to lipid ratio represents the presence of cancer. Here in panel (b) it is validated with the H&E staining where the cancer image is highly stained.^5^ Reprinted with permission from ref (5). Copyright 2018 Nature.

A label-free in situ intraoperative cancer detection system based on high wavenumber (2000 to 4000 cm^–1^) Raman spectroscopy was developed by Leblond and team for the detection and biopsy of brain cancer. They validated the integrated core needle biopsy system with an animal study on swine. Further, a human clinical trial was also conducted on 19 patients to identify the capacity of the newly devised HWN RS to decide between normal and cancerous brain tissues. The tissues were classified into dense (>60%), infiltered (5–60%), and normal (<5% or no) cancer cells to check the tumor border too as shown in Figure 4.^5^ Fifty-nine patients, 223 tissue samples, and 1273 spectra were obtained to differentiate meningioma and dura mater using Raman spectroscopy. They were analyzed with the help of a machine learning-based classifier which gave about 100% and 93.97% in the external set while the internal 5-fold cross-validation set gave 96.06 ± 0.03% and 95.44 ± 0.02% sensitivity and specificity, respectively. The study used the tissue samples obtained intraoperatively without any preprocessing and were measured using Raman spectroscopy within 20 min of excision to match the in vivo sample condition as much as possible. Though the sensitivity is obtained to be 100% from the classifier, single spot measurements cannot be used for diagnosis as in areas of infiltration the tumor margin in the tissue varies from point-to-point necessitating the need for more spectra.^31^

The feature engineering approach opted for by Leblond et al. analyses a data set of 547 spectra based on about 30 parameters through a Bayesian framework. This comprehensive molecular profiling infers plausible changes noticed in glioma like a nucleic acid increase, collagen IV overexpression, and a shift in the spectra of peaks involved in primary metabolism.^9^ A feature-driven Raman analysis was established by Stables and colleagues to ease and improve the real-time intraoperative use of Raman spectroscopy. They mapped the sub-band spectra with a frequency modulator (FM) to provide the output as sound signals varying for normal and disease conditions. Then participants were asked to predict based on listening tests. The classification accuracy was 71.1%, though feature extraction by SVM output was 88.99%. The parameters like hearing sensitivity and effects due to age and sex would also have significantly contributed to the results. Still, improvement in the sonification parameter might give better results.^32^

Straehle et al. interpreted ex vivo brain tumor samples via stimulated Raman histology (SRH) with the help of a novice neuropathologist to determine the accuracy and comprehensibility of SRH. The results were also compared with the same section’s hematoxylin-eosin (H&E) staining results. In terms of accuracy, SRH did not have a significant impact but was not inferior to that of the H&E stained sections as their accuracy stood at 87.3% against 88.9%, respectively. The ability of SRH images to highlight putative axons, tumoral, and glial fibers unlike in H&E staining talks about its comprehensiveness.^33^ Also, the SRH can be used as an intraoperative technique during surgeries as a parallel tool to that of the conventional due to their time-saving ability and easy processing of data due to their digital nature.^34^ Another study by Pekmezci and the group also showed similar results between SRH and H&E stained sections. The results were noninferior as both SRH and H&E stained groups confirmed only around half the samples for glioma infiltration at the tumor margin, while immunohistochemistry results were comparatively more significant than the other two.^35^

Odion and group explored the ability of SESORS in three different systems and offset values namely 4 mm in paraffin film and tissue phantom and 5 mm in a Macaque monkey skull. The system used for surface enhancement was a Gold nanostar with PEG and Raman active dyes. The group came up with a two-layer phantom setup that can give clean spectra by scaled subtraction. The top layer of the setup had DTTC-labeled nanostars while the bottom had Cy-7 labeled nanostars. After this, they went one step ahead to rectify the limitations in SESORS in terms of penetration of the skull, that too with good intensity. Further, as the penetration was to be through bone, which usually overwhelms underlying signals because of the phosphate groups, the authors used an axicon lens which has better area coverage with a better permissive laser. This modified setup was called inverse SESORS. This shows the potential of the novel system to be used in enhanced noninvasive brain cancer detection studies.^36^

**Figure 5:**
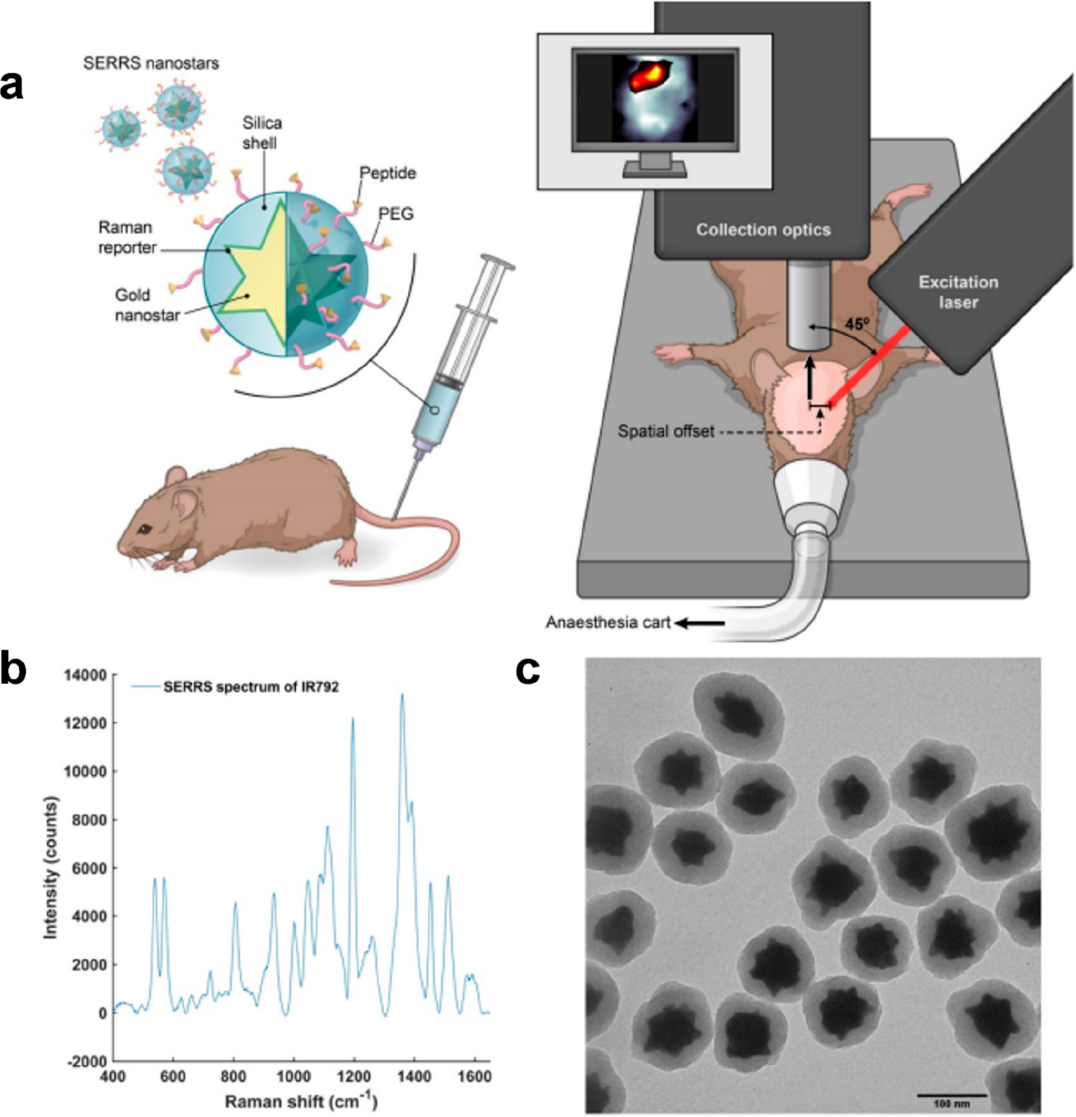
Surface-enhanced resonance Raman Spectroscopy (SERRS) system. Plasmonic gold nanostars (surface enhancer) bioconjugated with other moieties along with the schematic of the Raman with an in vivo animal operating setup (a) to give enhanced SERRS spectra (b). TEM image of gold nanostars (c).^37^ Reprinted with permission from ref (37). Copyright 2019 Ivyspring International Publisher.

An earlier study was also on similar lines. Moody and co-workers checked the potential of SESORS to noninvasively detect neurotransmitters inside the skull region from outside. The animal model they opted for was a cat skull as it has a skull thickness of 2 mm (humans: 3–14 mm), thus making the offset value to be 2 mm. They used Au as the surface-enhancing agent and coated it with neurotransmitters. These neurotransmitters namely serotonin, melatonin, and epinephrine were detected by SESORS at a minimum concentration of 100 μM.^38^ Nicolson and colleagues developed a (SESO(R)RS), “surface enhanced spatially offset resonance Raman spectroscopy”, to study glioblastoma in a noninvasive method. In this study, the group did in vivo imaging of deep-seated GBM in mice through the intact skull. They developed their own spatially offset Raman spectroscopy (SORS) system, integrated with the surface-enhanced Raman spectroscopy (SERS). The surface enhancement involved was a gold nanostar and a Raman-reporter dye system functionalized with cRGDyK peptide, as seen in Figure 5. The SORS was initially compared with conventional Raman spectroscopy against a PTFE phantom at 2 mm and 3 mm spatial offset while the SESO(R)RS system had an offset of 2.5 mm. This was not optimized further as the aim of the study was to visualize and interpret SESO(R)RS in Glioblastoma noninvasively.^37^

The molecular and electron level changes taking place between the peak 1584 cm^–1^ representing the cytochrome c and complex IV inhibits the controlling mechanisms of the electron transport chain. The results do not support the widely accepted Warburg mechanism for cancer but oxidative phosphorylation. Though they are the mechanism of action for normal cells, in cancer cells, there is an increased concentration of cytochrome c to regulate bioenergetics via ATP and also enhance denovo lipid synthesis. In conclusion, the team recommends Raman spectroscopy to continuously examine the redox state changes in the mitochondrial cytochrome and thereby malignancy of brain tumors.^39^ Zhou and colleagues studied glioma in 21 specimens using confocal Raman microspectroscopy in the visible range and found two new peaks increasing in intensity with increasing grade of glioma. One at 1129 cm^–1^ is attributed to phosphatidic acid, an unsaturated fatty acid, or lactic acid, which plays an important role in glycolysis and 1338 cm^–1^ to adenosine triphosphate (ATP). Both contribute to characteristic effects in the metabolism of cancer, especially the Warburg effect hypothesis,^40^ contradictory to ref (39).

The work involves the use of Raman Spectroscopy to compare, analyze, and understand Glioma-like stem cells that are either radiation resistant or intermediate or sensitive based on their IC50 values. The results demonstrated high intensities for nucleic acid bands. This was attributed to the higher proliferation rates seen in neurospheres in the order of resistant > intermediate > sensitive, appropriately affirming previous literature.^41^ Additionally, the glycogen and cholesterol levels were low in cancerous groups when compared to normal. The quantitative data were analyzed using CDA and then were measured in MGG23, MGG4, and SK1035 neurospheres. These were matched with their results in a clonogenic assay, and a minimum of 95% accuracy was obtained. The group also treated the GSCs with drugs like 2-DG, ZOL, and HC-3 and checked their Raman spectral bands to learn about their drug efficiency in treating cancer.^42^

A compilation of the results from various Raman studies irrespective of surgery or diagnosis or monitoring, their precision levels and the methods used to come to an inference quantitatively and qualitatively are presented in Table 2.

**Table 2:** Performance of Raman Spectroscopy against Brain Tumors

brain tumor sample	data analysis	sensitivity %	specificity %	accuracy %	highlight/observation/inference	ref
4 patients, 4 tissue samples: glioma (grades III and IV)	PCA-LDA			avg: 92.5	peaks associated with cytochrome c, RNA, and DNA are higher in cancer cells	(12)
90 tissue samples: meningioma (grades I and II)	PCA-QDA SPA-QDA	85.7	100	96.2	peaks of amino acids, CH_2_ deformation and bending phospholipid structural changes, amide III and C=O stretching differentiates between grades I and II of meningioma	(10)
	MCR-ALS					
	SPA-LDA					
	SPA-SVM					
	PCA-LDA					
	GA-LDA					
	PCA-SVM					
	GA-SVM					
	GA-QDA					
8 healthy samples and tumor samples from 5 patients (2 anaplastic astrocytoma and 3 glioblastoma)	PCA			96	SERS-specific spectra clearly discern healthy and cancerous brain tissue samples	(43)
Untreated, 2-DG, Zol, and HC-3 treated glioma stem-like cells (GSCs)	CDA			86.1	radiosensitizing ability of 2-DG, Zol and HC-3 (explored for the 1st time) for a radiation dose of 8Gy are studied	(44)
	PCA-LDA					
	LOOCV					
117 FFPE blocks, 59 glioblastoma tissue samples, 53 patients: necrosis, peritumoral zone, and vital zone identification	SVM	64	82	70.5	classifies tumor margin, thereby identifying normal and tumor environment	(45)
	5-fold cross-validation					
280 spectra, 19 patients: glioma (grades II–IV)	SVM	80	90	84	1st swine brain biopsy model	(5)
	LOOCV				human glioma surgery using modified hand-held Raman	
Human medulloblastoma (Daoy cells), γ radiation	PCA				compares biochemical changes shown in label-free (Raman) and labeled techniques (oxidative stress and metabolic activity) in medulloblastoma	(25)
	RMSECV					
Control	PLS-DA	77.1	83.4			
2 Gy		86.6	71.3			
10 Gy		74.1	88.0			
Immune cells grown on media conditioned by glioblastoma stem-like cells (GSCs)	PCA-LDA	>70	>70		CD73 and ZEB1 influence monocyte and T-cell phenotypes	(24)
	SVM	>67	>67			
	random cross validation using PLS					
1273 spectra, 223 samples, 59 patients (meningioma and dura matter)	SVM				high intensity of collagen in dura mater than in meningioma	(31)
	external test set validation	100	93.97			
	5-fold cross-validation	96.06	95.44			
3450 spectra, 63 fresh samples: normal and glioma (grades II–IV)	random forest (RF) and gradient boosting trees (GB)			83	reduced lipid content in the healthy group; increased DNA content in the tumor group	(7)
	LOPOCV					
	5-fold cross-validation					
121 samples						(19)
Normal tissues vs Brain tissues	SVM	100	71		normal healthy brain tissue concentration of lipid:protein is 1.15:1	
Healthy brain tissues vs Glioma tissues	SVM	100	96.3	99.6	grade IV glioma tissue concentration of lipid:protein is0.82:1	
						
Low grade (grades I and II) vs High grade (grades III and IV)	LOOCV	96.3	53.7	84.1		
11624 spectra, 73 samples - fresh healthy brain tissues vs glioma tissues	PCA-LDA	96	99	99	RS of 5-ALA-induced fluorescent samples outperforms RS analyzed samples	(46)
	LOOCV					
8 patients, 7 brain cancer types	PLS-DA	90	50		carotenoids, cytochrome c, fatty acids and protein peaks are the major Raman signals for classification	(47)
44 cancer patients, low vs high spectra	SVM	89	90		higher spectral levels increase sensitivity and specificity by 20% and 12%, respectively	(22)
	5-fold cross-validation					
Normal stem cells vs Inherent GSCs	PCA-LDA and CDA			99.6	resistant phenotype was reversed using small molecule inhibitors	(42)
Normal stem cells vs Radiation-induced	LOOCV			97.9		
Normal vs Tumour tissues in cerebellum (14 samples)	LDA			93.3	concentration of 16 biochemical compounds present in the brain was assessed using RS	(48)
Normal vs Tumour tissues in whole brain (28 samples)	PLS-DA			94.1		
Grade IV medulloblastoma vs Healthy tissues	PLS-DA	98.5	96		high levels of β sheet and low levels of α-helix conformation changes are seen in tumorous groups	(14)
	cross validation	96.3	92			
62 patients genetic subtypes of glioma	PCA-LDA	79–94	90–100		classifies IDH mutant, IDH wildtype astrocytomas, and 1p/19q codeleted IDH mutant oligodendroglioma	(27)
only IDH mutation	LOPOCV	91	95			
104 patient blood serum samples (grades I and II)	MLP				phenylalanine associated with brain metastasis of lung cancer	(49)
	RNN					
	CNN					
Glioma vs Control	PLS-AlexNet			100		
Glioma vs Lung cancer	5-fold cross-validation			95.2		
209 patients - fresh samples	PCA				astrocytoma IDH-1 mutant and oligodendroglioma, which varies only by 1p/19q codeletion, is identified with 81% accuracy	(26)
Non-neoplatic				100		
Primary GBM				90		
Recurrent GBM				100		
Astrocytoma				86		
Oligodendroglioma				90		
Metastasized glioma				90		
IDH mutated in oligodendroglioma and astrocytoma				81		
435 spectra, 19 tissue samples of brain cancer	DFA training set			95.1	6-year study on brain tumor classification using RS	(29)
	testing set			88.9		
10 patients - necrosis tissue from brain tissue (both tumorous and nontumorous)	PCA	84	89	87	Raman optimized by changing parameters like laser power, integration time, and signal-to-noise ratio to precisely identify necrosis tissue	(50)
						
12 patients: benign vs infiltered	SVM	86	90.1	88.3	differentiation of benign and tumor-marginalized areas using RS was done	(51)
133 spectra, 20 patients: Glioma	LVQ			89.5	introduces a new discrimination and classification system to predict the efficiency of RS	(52)
Normal white matter				85.7		
172 spectra - neoplastic vs normal brain tissues	PCA-Euclidian distance	97.4	100		variation in fatty acids, proteins, and hemoglobin was noticed between the meninges and cerebellum	(53)
						
1951 spectra of tissues					meta-analysis of the accuracy of RS in distinguishing cancer and normal tissues	(54)
Glioma		96	99			
Meningioma		98	100			
98 spectra - mouse model normal cells vs glioma cells	PCA	98.3	75		tumor discrimination accuracy of cells is better than tissue samples	(55)
						
						
8 patients - normal vs cancer cells	boosted tree	94	90	86	detected cancer tissues 1.5 cm beyond the detection level of MRI, in real-time	(56)
						
161 spectra, 17 patients	LOOCV				multimodal RS with MR guidance for real-time detection was developed	(57)
Dense cancer cells (>90% cancer cells)	boosted tree	97	91	93		
Infiltered cancer cells (≤90% cancer cells)	boosted tree	89	91	90		
22 specimens - normal brain tissue vs brain metastasis	PLS-DA			training set 97.1	primary tumors of brain metastasis were from colon, bladder, mamma, renal, prostate, and lung carcinomas	(58)
	linear SVM			99.5		
	radial SVM			99.8		
	PLS-DA			independent 70.4		
	linear SVM			80.3		
	radial SVM			89.3		
	KMC					
	VCA					
	5-fold cross-validation					
649 spectra, 64 samples, 28 pediatric patients	SVM				1st study to differentiate pediatric brain tumors from that of normal ones	(28)
Normal brain				96.9		
Glioma				96.7		
Medulloblastoma				93.9		
Ependymomas: high vs low grade	LOOV	100	96			
Normal tissue vs Low-grade glioma		91.5	97.8			
43 RR spectra from 7 brain cancer patients	SVM	90.9	100		a peak at 1572 cm^–1^ (amide II) was observed in all samples under resonance Raman spectroscopy but not in nonresonance spectroscopy	(59)
	ROC					
	PCA					
48 FFPE samples from 41 patients -normal, glioma, and metastatic samples	feature-driven SVM	mean 91.36	mean 96.19	97.01	accuracy of feature-driven SVM increased by 26.25% to that of conventional SVM	(32)
	feature-driven KNN			91.02		
	feature-driven LDA			95.38		
	10-fold cross-validation					
17 patients: normal vs cancer	LOOCV				effect of room light artifacts was explored using artificial neural networks (ANN)	(60)
(Excluding light artifacts)	boosted trees	93	91	92		
	ANN	94	89	92		
(Including light artifacts)	boosted trees	84	51	71		
	ANN	91	89	90		
952 spectra, 48 FFPE samples, 41 patients	Cross validation				metastatic set had reduced 718 and 925 cm^–1^ and higher 1250, 1400, and 1670 cm^–1^ peaks compared to normal and GBM sets	(18)
GBM	PCA-LDA	100	94.44			
Metastatic brain		96.55	100			
Normal brain		85.71	100			
						
31 spectra, 8 patients : normal vs brain metastases (lung)	SFS-SVM	85	75		augmented RR peaks of tryptophan, lactate, and amide II are seen in brain metastases	(61)
						
8 patients: normal vs lung cancer metastasized in brain	PCA-LDA	97	100		in metastases, amide I and collagen have high intensity; lipids and lipoproteins have low intensity, while it is vice versa in normal tissue	(11)
95 spectra, 45 tissue samples: GBM, necrotic, gray matter	DFA				gray matter showed higher lipid content and necrosis, higher protein and nucleic acid content, while GBM remained in the middle	(62)
Training				99.6		
Validation of fresh				97.8		
Validation of FFPE				77.5		

## 6 Multimodal Raman Spectroscopy

An intraoperative study comparing the use of 5-ALA-induced fluorescence-driven surgery and the possibility of replacing it with Raman was explored. With 5-ALA fluorescence-guided surgery, surgeons can switch from white light to blue light with a click of a button which is not possible if Raman spectroscopy is integrated into surgery. But 5-ALA fails in terms of successfully representing the tumor margins, whereas Raman spectroscopy manages to be efficient both at the core and margin of the tumor tissue and obvious with the normal tissues. Clearly, the Raman results upheld the previous literature as peaks of lipids (825, 853, 1087, 1124, and 1305 cm^–1^) and DNA (517, 885, 1206, and 1342 cm^–1^) are dominant in glioma groups, while amino acids (540, 615, 635, and 649 cm^–1^) and amide III protein (1522 and 1553 cm^–1^) peaks are dominant in normal brain tissues. Thus, the authors conclude by suggesting the combined multimodal use of both 5-ALA fluorescence and Raman spectroscopy to target cancer tissues in vivo.^46^ Another intraoperative study by Jermyn and group investigated the detection ability of Raman beyond the preoperative MRI detection levels. Their results supported Rahman’s discrimination capacity as it was able to discern low-density cancer 1.5 cm beyond the MRI levels.^56^ The same team, in another similar work, compared the better of MRI and Raman imaging for cancer detection in depth with specifics. It was found that Raman outperformed T1-contrast enhanced MRI and T2-weighted MRI by detecting invasive cancer cells to a maximum of 3.7 and 2.4 cm beyond T1 and T2 levels, respectively. Also, Raman spectroscopy was able to find as small as 6 invasive cancer cells for each 0.0625 mm^2^.^63^

Gajjar proposes the complementary use of both FTIR and Raman for brain tumor diagnosis as they observed 1045 to 1545 cm^–1^ (phosphate to carbohydrate) changes in high-grade gliomas and alteration in 1121 to 1020 cm^–1^ (RNA to DNA) ratio in meningioma, only with FTIR. Similarly, changes in the 1670 to 1001 cm^–1^ (cholesterol to phenylalanine) ratio to discriminate low-grade astrocytoma from meningioma were observed only in Raman.^64^ But another similar study by Depciuch and colleagues comparing tumorous and nontumorous brain tissues is profiled based on their molecular changes in FTIR and Raman spectroscopy. The findings infer major changes in the sphingomyelin and phosphatidylcholine levels when analyzed via Raman. On the other hand, FTIR only on Kramers–Kronig transformation was able to identify changes in 1450, 2847, and 2915 cm^–1^, all belonging to lipid vibrations, but the same was more comprehensive in Raman.^65^

Combined spatial frequency and resonance Raman spectroscopy was developed by Zhou et al. to distinguish between brain metastases and normal brain tissues. The SFS’s dominant amplitude moved away from the center in all directions, indicating low spatial frequency in the center and vice versa. The higher frequency low amplitude system prevailed majorly in cancerous tissues contrasting to normal tissues. The RR spectra results revealed the significant intensity augmentation of lactate, tryptophan, and amide II. The carotene peaks were either constant or moved to a higher frequency in metastases tissues. These results on further interpretation with SVM showcased reliable similarity with the gold standard “histopathology”.^61^

## 7 Nonlinear Raman Spectroscopy

In general, nonlinear Raman spectroscopy involves more than one laser source, and the Raman signal is independent of incident light intensity unlike in conventional, linear Raman spectroscopy. The interaction of the energy from different laser sources, usually a pump beam and stokes beam, enables a high signal-to-noise ratio (SNR) and resolution magnitude compared to spontaneous Raman spectroscopy. Widely used nonlinear Raman spectroscopy techniques are coherent anti-Stokes Raman spectroscopy (CARS) and stimulated Raman spectroscopy (SRS).

### 7.1 Coherent Anti-Stokes Raman Spectroscopy (CARS)

Uckermann with his team explored the ability of CARS to delineate glioblastoma, brain metastases of breast cancer, and melanoma in a mouse model. The CH-tuned CARS setup showed lower intensity in their signals, unlike normal tissues because lipid content is lower in tumor conditions. The intensity of reduction was not as significant in metastases as that of glioblastoma. This was attributed to the primary brain cancer characteristic in GBM compared to the metastases of melanoma and breast cancer, thereby helping in the discernment and diagnosis.^66^ Pohling et al. mapped the tumor region in a mouse brain tissue using multiplex-CARS. The infiltration region was detected by SVM. Unlike most other works, which color codes based on spectral intensity, this study color-coded based on pathological information. The final output was checked with the reference H&E stained samples and was found to be in-line with them.^67^ A study by Galli and group investigated the effect of tissue fixation methods on CARS images and tissue biochemistry. The results revealed that fixing using methanol-acetone lowers the lipid content making it incompatible for CARS, while formalin did not alter the biochemistry much nor the contrast and intensity of the images obtained.^68^

Most CARS studies are done with a multimodal approach. A novel multimodal photon microscopy using nonlinear imaging was put forth by Meyer and team to replace or complement the current gold standard “histopathology”. The multimodal setup involves coherent anti-Stokes Raman spectroscopy (CARS), second harmonic generation (SHG), and two photon-excited fluorescence microscopy (TPEF). Only the aliphatic CH_2_ band is used by CARS to discern tumor and normal tissue, while SHG gives high chemical selectivity, especially to the blood vessels, arachnoid membrane, and other components rich in collagen. On the other hand, TPEF helps in imaging morphology of the tissue label-free. The results combined overall were also verified with FTIR and Raman spectroscopy. This completed setup managed to produce similar results to that of H&E staining but only on a large scale. At a single-cell level, the latter was superior.^69^ Study by Galli et al. involved green fluoroscence protein (GFP) tagged to glioma cells and 5-ALA for targeting glioma both used by TPEF and CARS, respectively, to demonstrate the identification of infiltrated brain tumors in both human and mouse models. The usage of CARS images with GFP helps in finding the neoplastic tumor in a single cell level, making it more precise and giving an idea about the cellular changes in both ex vivo and in vivo glioma.^70^ Another similar study used CARS and TPEF images of 55 brain tumor lesions and overlaid them to give a highly resolute image comparable to that of the gold standard histology images. The CARS system used a 670 nm laser as its pump beam and a 830 nm Ti:Sa beam at its Stoke’s beam source. These were recombined spatially and temporally to come up with the CARS images. Thus, the images obtained can guide neurosurgeons to avoid brain hemorrhage as the images were able to represent even the blood vessels while also saving time for neuropathologists to identify section regions minimizing the invasiveness of the surgery.^71^ One more study by Uckermann et al. utilized CARS and TPEF combination to classify between brain tumor, its subtypes, brain metastases of other solid tumors, and nontumors. Samples were obtained from 382 patients and 28 nontumor brain samples. The texture analysis done using CARS was further analyzed with LDA. The developed system was able to identify all nontumors with 100% accuracy, while the overall correct rate stood at 96%. Forty-two samples were analyzed in a fresh state to test the system’s ability to translate to the clinic. Also, the setup was able to classify tumors even with an image resolution of 1 μm.^72^

### 7.2 Stimulated Raman Scattering Spectroscopy (SRS)

**Figure 6:**
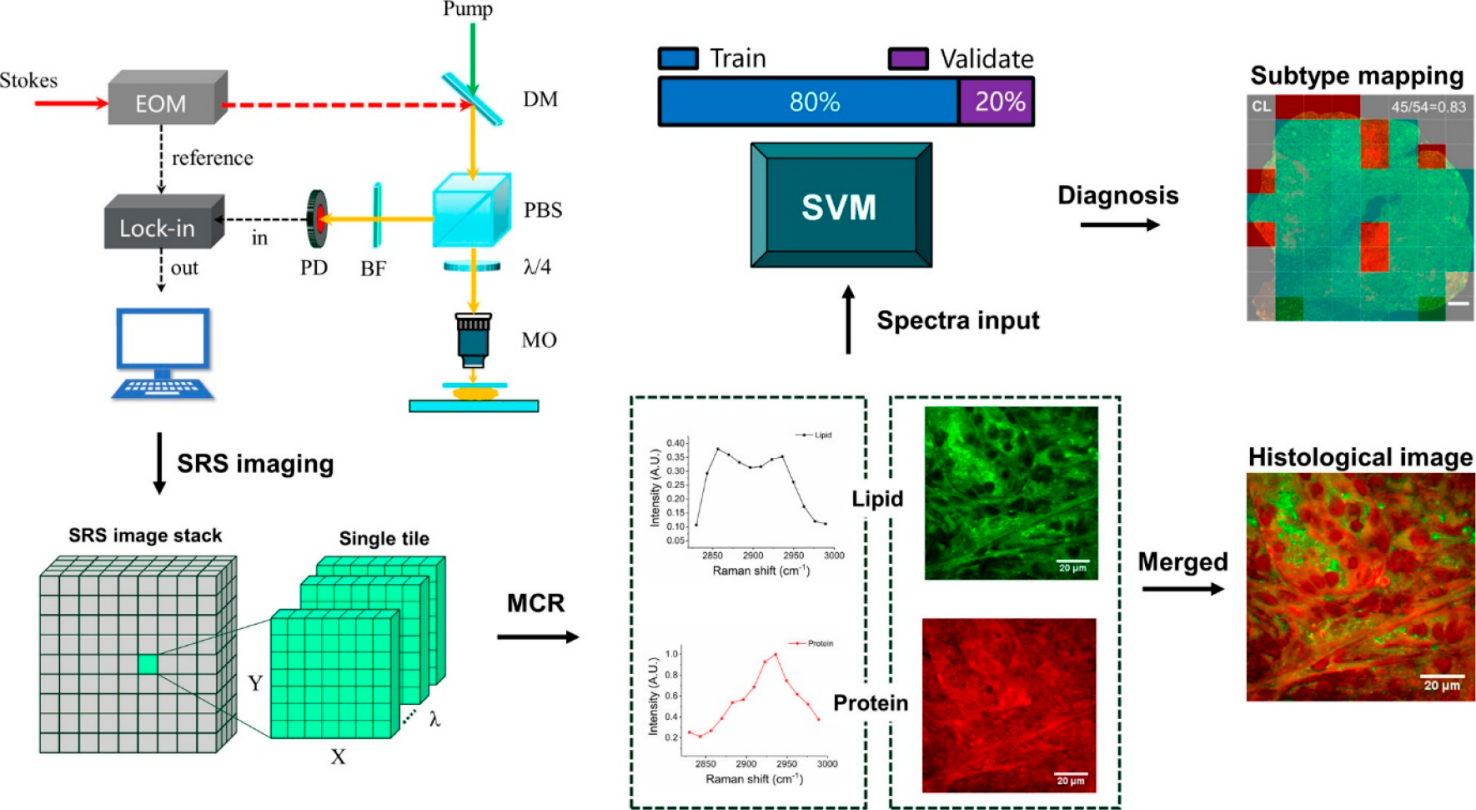
Graphical representation of SRS imaging diagnostic platform for rapid glioblastoma subtyping. Here, the image stack denotes the tissue area obtained under an SRS microscope. Each tile of the stack undergoes multivariate curve analysis to express the major spectral components. Among those components, the decomposed spectra are used for subtyping glioblastoma while reconstructed concentration maps are merged to give histological images.^74^ Reprinted from ref (74). Copyright 2021 American Chemical Society.

Ji et al. tapped the potential of SRS to discriminate between neoplastic and non-neoplastic in both ex vivo and in vivo mouse models. The team used a tunable pump beam of 600 to 1000 nm from an optical parametric oscillator and a Stoke’s beam of 1064 nm over the sample. The energy difference between the two beams was matched with the molecular vibrations to induct SRS. The results acquired through SRS were matched with that of H&E staining, and a Cohen’s κ value of 0.98 was obtained, implying an approximate 98% match.^73^ The SRS imaging system integrated with multivariate curve resolution (MCR) and quadratic SVM was utilized by Bae and colleagues to virtually do the H&E staining in glioblastoma specimens and further subtype classification, where illustration of the same is seen in Figure 6. Apart from visualizing the vascular proliferation and demyelination progress, the setup was able to signify the intratumoral heterogeneity to a certain extent.^74^ One study employed fiber-laser-based SRS for histology. The aim of the study was to explore the competence of SRS to identify the tumor attained from the infiltrate tumor margins of brain tissue samples. The stimulated Raman histology (SRH) and H&E stained samples with a residual tumor were identified properly in 49% of the samples, while the same for immunohistochemistry (IHC) was 56%. The observations were done by three neuropathologists in a blinded manner.^35^ In another work by Ji et al., fresh, unprocessed samples from 22 brain cancer patients was obtained to detect the tumor infiltration. The team exercised quasi-likelihood strategy to develop a generalized additive model (GAM) exploiting 1477 field of view (FOV) images from 3 epilepsy and 15 brain cancer patients. Half the set of images were used to train the model, while the remaining were used to test it. Due to SRS’s proficiency to recognize the histoarchitectural structures, axonal, and cellular densities unlike other Raman systems, they were inculcated in the classifier to improve the result leading to a specificity of 98.5% and sensitivity of 97.5% and also a κ = 0.86 against the H&E stained light microscopy.^75^

A multimodal SRS-SOCT (spectroscopic optical coherence tomography) setup was exploited by Soltani and group to assess its capability to distinguish between a normal and tumorous brain in a 9 L gliosarcoma rat model. It was found from the work that SOCT can resolve spatial and spectral features of the SRS comparatively easily which leads to faster data acquisition of even larger regions, which makes the multimodal setup a good option to consider for clinical use.^76^ Orringer et al. were the first ones to come up with a SRS microscopy for intraoperative use in the clinic and also SRH, virtual H&E staining. The virtual stained images were obtained with 2 s per frame FOV in a mosaic pattern, stitched, and recolored, taking only 2.5 min for a whole mosaic. The instrumental setup consisted of a portable fiber-laser-based microscopy integrated with the SRS. Samples from 101 brain tumor patients were considered for the study, with half done via frozen sectioning and the remaining via SRH. The portable setup showed a 25-fold enhanced SNR. The inter-rater reliability [Cohen’s kappa (κ)] consistently remained above 0.89 in all cases whether it be distinguishing between glial and nonglial, SRH and H&E staining, or lesional and nonlesional with an accuracy of above 90%.^77^

## 8 Chemometrics

Aguiar et al. imaged 263 spectra of various brain tissues both tumorous and nontumorous and analyzed 16 biochemical compounds by using a least-squares fitting spectral model. The discriminant models used were LDA and PLS-DA. The accuracies for separating normal and tumor models were 93.3% and 94.1%, respectively, which are approximately the same. This suggests the use of any one of the models for discrimination analysis in the future.^48^ The aim of the study was to discriminate between grades I and II meningiomas via Raman microspectroscopy with utmost accuracy. For the same, the team investigated 90 meningioma samples and the biochemical changes were analyzed with multiple chemometric methodologies. Among them, SPA-QDA and PCA-QDA showed higher accuracy values of about 96.2% with sensitivities and specificities at 85.7% and 100%, respectively.^10^ Lilo and team collected 95 meningioma tissue samples from grades I and II combined. The samples were analyzed using Raman hyperspectral imaging and processed by 3D-PCA-QDA. The results of postprocessing showed 96% accuracy with 95% specificity and 100% sensitivity.^78^

Chen and colleagues used different computational algorithms to find the most accurate prediction among convolutional neural networks (CNN), recursive neural networks (RNN), multilayer perceptron (MLP), and AlexNet for binary classification between control groups and glioma and glioma and lung cancer using 5-fold cross-validation. AlexNet’s accuracy stood out at 100% and 95.2%, respectively, making it the best algorithm among the ones used.^49^ Jermyn et al. used artificial neural networks to accommodate the Raman spectroscopy to be used in the presence of light artifacts to detect invasive cancer cells, thereby enhancing clinical translation ability and robustness for intraoperative use. The team was able to achieve an accuracy of about 90%.^60^ Liu and group proposed a new method for the data analysis of Raman spectra, which was previously commonly used in pattern recognition. It is a neural network-based algorithm and is called learning vector quantization (LVQ). The normal and glioma tissue diagnosis accuracy was 85.7% and 89.5%, respectively.^52^

An SVM algorithm was introduced with a 2-level discriminant analysis to predict the primary tumors of brain metastases. The results obtained via radial-SVM surpassed that of linear-SVM and PLS-DA. The algorithm prediction was 99% accurate in successfully identifying the independent primary tumors.^58^ In another study, three classifiers (LDA, KNN, and SVM) went through both principal components and sub-band feature extraction to improve the classification efficiency of Raman spectroscopy. In this modified feature-driven classifier setup, LDA negatively performed with a 1.16% reduction in efficiency, but the other two classifiers KNN and SVM improved by 25% and 26.25%, correspondingly.^32^ One machine learning algorithm used was developed using Scikit-learn to classify performance with leave-one-patient-out cross-validation: gradient boosting trees and random forest.^7^

## 9 Conclusion

The review clearly establishes Raman to be a potential method in the detection, monitoring, and surgery of brain cancer. This can go as a standalone tool to improve the lives of brain tumor patients in the future. This can also proceed as a multimodal means along with conventional imaging instruments like the MRI and CT scanning techniques. Unlike histopathology which requires hours of time and expert neuropathologists to analyze, Raman spectroscopy can provide unbiased accurate interpreted data in real-time. It also does not need any preprocessing or staining.^28^ Though currently, there are not any Raman-approved surgery for brain cancer, the idea of it becoming reality is not far-fetched. With setups like SERS which require surface enhancers, the surface enhancer can be made with a smart nanoparticle system thereby they become either pH, temperature, or photosensitive so that interaction with a laser can ablate the tumors. But, to attain these sooner, there needs to be even wider human clinical trials considering as many parameters as possible to bring it to the clinic for use. Further, more accurate ML tools are necessary to interpret the data precisely. So, the results can be used a step further to help in personalized medicine and decide on a regime of treatment based on the biomolecular signatures observed.

## 10 Limitations and Future Prospects

A simple web-search on the FDA clinical trials site for the term Raman spectroscopy shows about 108 clinical studies as of mid-June 2023, including completed, withdrawn, currently active, and recruiting. Of these, only 2 studies one completed and one recruiting are related to the brain but that too not of brain cancer.^79^ This clearly shows the need for more clinical trials using Raman spectroscopy as different modalities. But this is due to few practical hurdles, like the interference of ambient light with/and fluorescence, Raman’s sensitivity to water. The major hindrance is the portability of the instrument, and real-time data analysis with minimal to no delay and high complexity of the instrument for a clinician to handle instigates the need for a Raman spectroscopy expert instead of an expert neuropathologist. So, this does not make much of a difference. And, of course, the cost of the setup has to come down many-fold. The future research in the field must concentrate on the miniaturization, simplification, and cost-effectiveness of the instrument without compromising on the safety and efficiency. Now, with the rise of AI, potent ML models should be developed to increase the accuracy of the results. Recently, Jiang et al. have come up with the first open data set of 1300+ clinical SRH images from more than 300 patients called OpenSRH.^80^ This kind of open public data sets and repositories, say a universal repository created by a consortium including academic, industry, and hospitals, can lead to enhanced collaborative research in Raman spectroscopy leading to a quicker progression toward affordable, safe, and efficient state-of-the-art Raman spectroscopy ready for clinical use.
